# The millipede genus *Caucasodesmus* Golovatch, 1985, with the description of a new species from the Crimea, Ukraine (Polydesmida, Diplopoda, Trichopolydesmidae)

**DOI:** 10.3897/zookeys.93.1159

**Published:** 2011-04-29

**Authors:** Sergei I. Golovatch

**Affiliations:** Institute for Problems of Ecology and Evolution, Russian Academy of Sciences, Leninsky pr. 33. Moscow 119071, Russia

**Keywords:** millipede, Trichopolydesmidae, taxonomy, new species, cave, Crimea

## Abstract

The hitherto monotypic genus *Caucasodesmus* is new to the Ukrainian list due to the discovery of *Caucasodesmus tauricus* **sp. n.** in a cave in the Crimea. The new species is easily distinguished from *Caucasodesmus inexpectatus* Golovatch, 1985, the type, and only other, known species of this genus, in the abundantly setose collum and following metaterga, and more elaborate gonopods. The status of *Caucasodesmus*, which shows in the superfamily Trichopolydesmoidea where it definitely belongs such evident generic-level apomorphies as the absence of bacilliform sensilla on antennomeres 5 and 7, of a cannula on the gonocoxite, and of a seminal groove on a biramous gononod telopodite (apparently, both latter characters are functionally correlated to each other), is refined by formally reassigning it to the family Trichopolydesmidae.

## Introduction

The millipede fauna of the Crimea has recently been reviewed (Golovatch 2008), with only 14 species from 11 genera, seven families and six orders being involved. Of these, only two or three species are possibly Crimean endemics, whereas most show more or less widely (Euro-)Mediterranean distributions.

The more so important is the present discovery of still another new millipede in a cave in the Crimea. Unlike all other faunal elements, this new species might prove to represent the first truly relict, palaeoendemic in the diplopod list of that peninsula.

Material serving as the basis for the present contribution was captured with pitfall traps, later transferred into 75% alcohol and is currently deposited in the collection of the Zoological Museum, State University of Moscow, Russia. Specimens were studied and illustrated using standard stereomicroscopic, photographic and drawing equipment.

## Systematics

**Trichopolydesmidae Verhoeff, 1910**

### Caucasodesmus

Golovatch, 1985

Caucasodesmus  Golovatch, 1985: 40.

#### Type species:

*Caucasodesmus inexpectatus* Golovatch, 1985, by original designation.

### Caucasodesmus tauricus sp. n.

urn:lsid:zoobank.org:act:66FEBC49-89EC-432E-A0E2-561179B19B24

http://species-id.net/wiki/Caucasodesmus_tauricus

Figs 1 8 

#### Type material:

Holotype ♂, Ukraine, Crimea, Mt Villya-Burun, Cave Villyaburunskaya, pitfall traps, 12.05.2008–12.10.2010, leg. A. Koval. – Paratype: 1 ♀, same locality, 19.07.2004–17.07.2006, leg. A. Koval.

#### Diagnosis:

Easily distinguished from *Caucasodesmus inexpectatus* Golovatch, 1985, the type, and only other, known species of this genus, by the abundantly setose metaterga and more elaborate gonopods.

#### Description:

Length of both sexes ca 8 mm, width of midbody pro- and metazona 0.8 and 1.5 mm, respectively. Coloration in alcohol from uniformly pallid to light yellowish.

**Figures 1–4.**
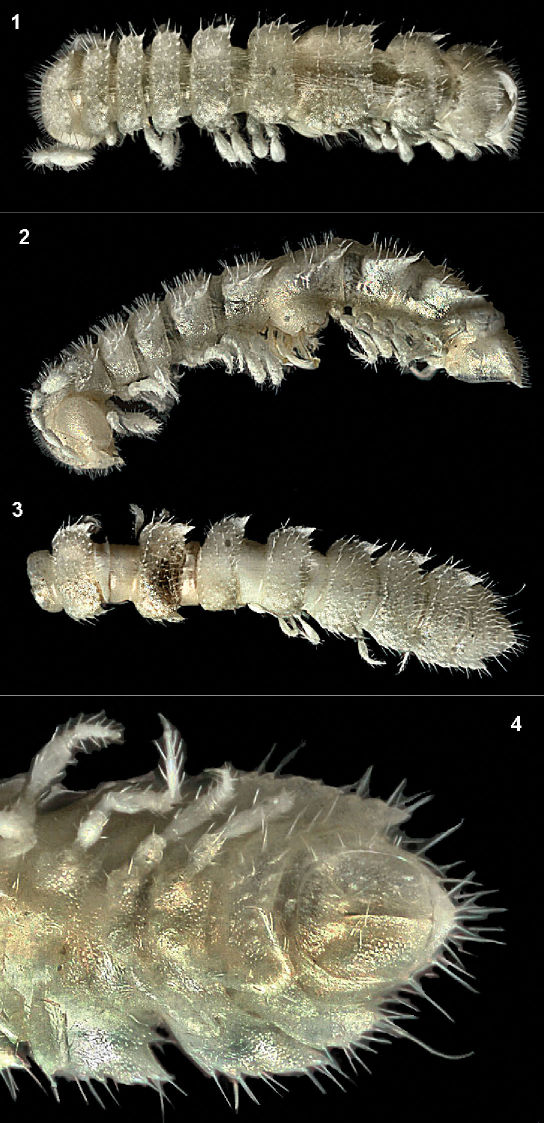
*Caucasodesmus tauricus* sp. n., holotype. **1, 2** anterior half of body, dorsal and lateral views, respectively **3, 4** posterior portion of body, dorsal and ventral views, respectively. Photographed not to scale.

Body with 20 segments. Tegument mainly dull, at most slightly shining, texture very delicately alveolate. Head densely pilose throughout; epicranial suture distinct but thin; isthmus between antennae ca 1.5 times broader than length of antennomere 1, still broader than diameter of antennal socket. Antennae rather short, evidently clavate due to a considerably enlarged antennomere 6, slightly overreaching segment 2 dorsally; antennomeres 2, 3 and 6 longest, subequal in length (Figs 1, 2); only antennomere 6 with a large, compact, roundish, distodorsal group of bacilliform sensilla.

In width, collum < segment 2 = 3 < head = 4 < 5=16 (♂) or head = collum = segment 2 = 4 < 5=16 (♀), thereafter body gradually tapering towards telson. Paraterga moderately developed, starting from collum, subhorizontal to slightly declivous, set high but always lying slightly below a faintly convex dorsum, devoid of shoulders frontally (Figs 1–3). Caudal corner of postcollum paraterga invariably spiniform, pointed, starting from segment 4 extending increasingly further than rear tergal margin. Lateral edge of paraterga with neither marginal groove nor thickening, with 5–6 clear setigerous indentations. Pore formula normal, ozopores evident, round, located laterally in front of caudalmost incision. Collum and following metaterga beset with numerous medium-sized setae set on minute knobs, polygonal bosses missing (Figs 1–3). Stricture between pro- and metazona wide, shallow and smooth. Limbus very thin, microdenticulate. Pleurosternal carinae absent (Fig. 2). Epiproct short, conical, directed caudoventrally; preapical papillae small (Fig. 4). Hypoproct subtrapeziform, setiferous papillae at caudal corners evident, rather well separated.

**Figures 5–8.**
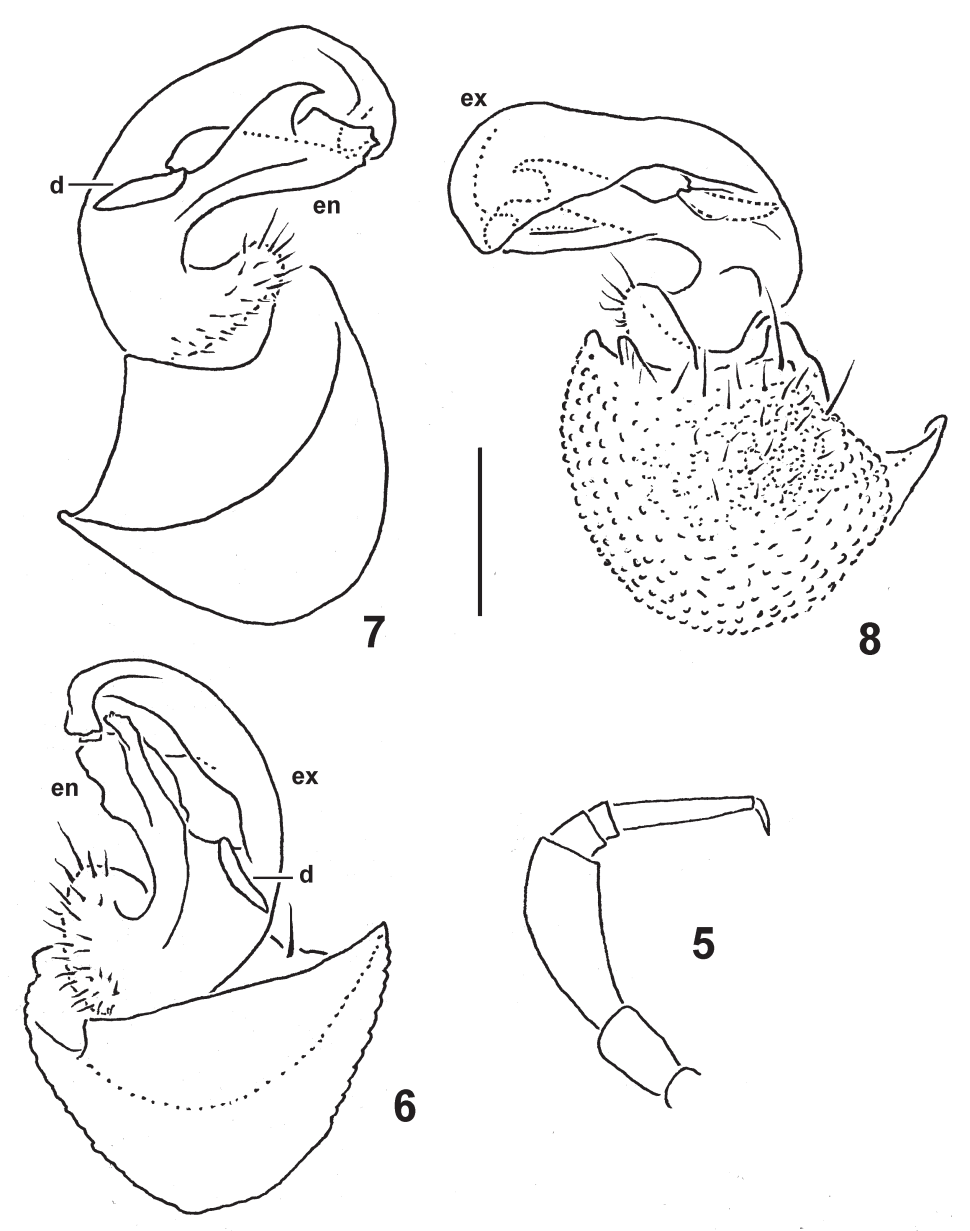
*Caucasodesmus tauricus* sp. n., holotype. **5** leg 9 (setae not shown) **6** rightgonopod, anteromesal view **7, 8**,left gonopod, mesal, and lateral views, respectively. Scale bar: 0.4 (**5**) and 0.2 mm (**6–8**).

Sterna without modifications, poorly setose. Epigynal ridge very low. Legs rather short (Figs 2, 5), ca 1.2–1.3 (♂) or 0.9–1.0 (♀) times as long as midbody height; ♀ legs slightly slenderer; ♂ legs with clearly enlarged prefemora and femora; tarsi especially long and slender, claw long, ca 1/4 length of tarsus; sphaerotrichomes missing (Fig. 5).

Gonopod aperture large, transversely oblong-oval, taking up nearly all of ventral part of metazonite 7. Gonopods (Figs 6–8) with large, globose, medially fused coxae carrying rather numerous setae laterally, but no trace of a cannula. Telopodite subfalcate, distally of a rather short prefemoral (= setose) portion split into two branches: exomere (**ex**) largest and longest, more simple, whereas endomere (**en**) shorter, more complex in shape; an evident, tooth-like, mesal process (**d**) at base between both **ex** and **en**; no trace of a seminal groove.

#### Remarks.

This species is an unquestioned relict troglobite and, based on its zoogeographical traits, might well represent the first palaeoendemic in the diplopod fauna of the Crimea.

Only one species of *Caucasodesmus* has hitherto been known: *Caucasodesmus inexpectatus* from Cave Nyvjin Lagat (= Nyvdzhinlagat, = Tagardonskaya) in North Ossetia, central Caucasus Major (Golovatch 1984/85). The second congener, *Caucasodesmus tauricus* sp. n., shares with the type species such remarkable, clearly generic-level apomorphies as the absence of bacilliform sensilla on antennomeres 5 and 7, of a cannula on the gonocoxite, and of a seminal groove on a biramous gononod telopodite. Apparently, both latter characters are functionally correlated to each other, differing from the loss of a cannula alone which is observed in the families Dalodesmidae and Rhachodesmidae. The differences lie in *Caucasodesmus inexpectatus* showing a far more moniliform body, only three transverse rows of setae on the collum and following metaterga, and less strongly elaborate gonopod telopodites.

## Systematic position of Caucasodesmus

Originally, *Caucasodesmus* was treated as a genus of the small Holarctic family Macrosternodesmidae (Golovatch 1984/85). However, Shear and Shelley (2007), in their recent reassessment of the Macrosternodesmidae, have ejected *Caucasodesmus* from that family, leaving it unclassified. These authors have also advanced a new terminology of the various parts of a polydesmidan gonopod. This has been further refined even more recently (Shear et al. 2009), in particular in accepting such denominations as exo- and endomere.

The superfamily Trichopolydesmoidea can be defined by its gonopod prefemoral (= setose) part orientated mostly transversely to the body’s main axis, extending mesally across the entire width of the coxae (Hoffman 1982; Simonsen 1990). Within this superfamily, where *Caucasodesmus* undoubtedly belongs, there are several, mainly small families in addition to Macrosternodesmidae: Trichopolydesmidae, Neoarctodesmidae, Furhmannodesmidae and Mastigonodesmidae. To find a new, more suitable place for *Caucasodesmus*, their diagnoses must briefly be reiterated, especially as regards their gonopod conformation.

Macrosternodesmidae (Shear and Shelley 2007; Shear et al. 2009): In this Holarctic family, the gonopod aperture is large, transversely oval. The coxae completely fill the respective halves of the aperture, excavated mesad to accommodate the telopodites; the prefemora are horizontal or angling ventromesad, giving rise to the acropodite and often, but not always, to an additional projection; the acropodite part distal to the origin of a solenomere (distal zone) variably configured, sometimes folded, flattened, and not recognizable as such; the solenomere is long and narrow, arising subterminally, with neither a hairpad nor an accessory seminal chamber (= ampulla); the seminal groove opens terminally.

Nearctodesmidae (Shelley 1994; Shear et al. 2009): This small Nearctic group shares basically the same gonopod conformation with Macrosternodesmidae. No wonder it has sometimes been treated as only a subfamily or even a possible synonym of the latter family.

Trichopolydesmidae (Ceuca 1958; Mauriès 1983): This small, mainly Mediterranean family shows basically the same gonopod structure as the previous two groups, except in the prefemoral part sometimes being shortened (e.g. *Galliocookia* Ribaut, 1955, *Occitanocookia* Mauriès, 1980 and a few others), i.e. rather strongly resembling the condition observed in the family Polydesmidae (see Hoffman 1980; Simonsen 1990), while the telopodite is bi- or uniramous, far less elaborate, often with a long flagelliform solenomere. Yet I am inclined to follow Mauriès (1983) in treating such somewhat deviating genera as representing rather peculiar Trichopolydesmoidea.

Mastigonodesmidae (Mauriès 1980, 1982): This very small, purely western Mediterranean group of polydesmidean millipedes is sometimes regarded as only one of the numerous genera of Polydesmidae (Hoffman 1980; Simonsen 1990), apparently because the gonopod prefemoral part in *Mastigonodesmus* Silvestri, 1898, is also shortened, but, due to its globose gonocoxae and a peculiar, parabasal, long and coiled solenomere, it seems more similar to trichopolydesmoids. So I am again inclined to follow (Mauriès (1980, 1982) in regarding this group as representing rather peculiar Trichopolydesmoidea as well.

Fuhrmannodesmidae (Golovatch 1994): This profoundly diverse, pantropical group of small polydesmideans shows a wide range of situations transitional in gonopod conformation between the typical Polydesmoidea and the typical Trichopolydesmoidea. At least in the Neotropical fauna, the gonopod coxae can be small and devoid of a gonocoel, with (sub)erect telopodites, yet more often the coxae are enlarged and deeply excavate for the accommodation of more stout, usually elaborate telopodites that can have a shortened to medially stretched prefemoral part supporting either crossing or parallel acropodites, the latter with or without a distinct solenomere. This highly heterogeneous assemblage certainly merits splitting into several natural families, but such a task is by necessity to be deferred because of the numerous genera and species involved, many of which still require revision.

Based on the above diagnoses and distributions, it appears to be quite difficult to unequivocally reallocate *Caucasodesmus*. The correlated absence of both a cannula and a seminal groove is probably a sufficiently strong apomorphy to erect still another family of Trichopolydesmoidea for the accommodation of solely this genus, but I refrain here from doing so pending more information becomes available. New taxa are still being regularly described, new synonymies established, and old types revised. Instead I reassign *Caucasodesmus* to Trichopolydesmidae as a family not only representing the oldest taxon in the superfamily, but also one which shows the same basic traits of gonopod structure and a coherent distribution pattern.

## Supplementary Material

XML Treatment for Caucasodesmus

XML Treatment for Caucasodesmus tauricus
